# Conquering hypertension in Vietnam—solutions at grassroots level: study protocol of a cluster randomized controlled trial

**DOI:** 10.1186/s13063-020-04917-8

**Published:** 2020-11-27

**Authors:** Duc A. Ha, Oanh T. Tran, Hoa L. Nguyen, Germán Chiriboga, Robert J. Goldberg, Van H. Phan, Cuc T. Nguyen, Giang H. Nguyen, Hien V. Pham, Thang T. Nguyen, Thanh T. Le, Jeroan J. Allison

**Affiliations:** 1 Ministry of Health, Hanoi, Vietnam; 2 Health Strategy and Policy Institute, Hanoi, Vietnam; 3 Department of Population and Quantitative Health Sciences, University of Massachusetts Medical School, 55 N Lake Ave, Worcester, MA 01655 USA; 4 National Heart Institute, Hanoi, Vietnam; 5 Vinmec Healthcare System, Hanoi, Vietnam

**Keywords:** Hypertension, Self-monitoring blood pressure, Storytelling, Trial, Vietnam

## Abstract

**Background:**

Vietnam has been experiencing an epidemiologic transition to that of a lower-middle income country with an increasing prevalence of non-communicable diseases. The key risk factors for cardiovascular disease (CVD) are either on the rise or at alarming levels in Vietnam, particularly hypertension (HTN). Inasmuch, the burden of CVD will continue to increase in the Vietnamese population unless effective prevention and control measures are put in place. The objectives of the proposed project are to evaluate the implementation and effectiveness of two multi-faceted community and clinic-based strategies on the control of elevated blood pressure (BP) among adults in Vietnam via a cluster randomized trial design.

**Methods:**

Sixteen communities will be randomized to either an intervention (8 communities) or a comparison group (8 communities). Eligible and consenting adult study participants with HTN (*n* = 680) will be assigned to intervention/comparison status based on the community in which they reside. Both comparison and intervention groups will receive a multi-level intervention modeled after the Vietnam National Hypertension Program including education and practice change modules for health care providers, accessible reading materials for patients, and a multi-media community awareness program.

In addition, the intervention group only will receive three carefully selected enhancements integrated into routine clinical care: (1) expanded community health worker services, (2) home BP self-monitoring, and (3) a “storytelling intervention,” which consists of interactive, literacy-appropriate, and culturally sensitive multi-media storytelling modules for motivating behavior change through the power of patients speaking in their own voices. The storytelling intervention will be delivered by DVDs with serial installments at baseline and at 3, 6, and 9 months after trial enrollment. Changes in BP will be assessed in both groups at several follow-up time points. Implementation outcomes will be assessed as well.

**Discussion:**

Results from this full-scale trial will provide health policymakers with practical evidence on how to combat a key risk factor for CVD using a feasible, sustainable, and cost-effective intervention that could be used as a national program for controlling HTN in Vietnam.

**Trial registration:**

ClinicalTrials.gov NCT03590691. Registered on July 17, 2018. Protocol version: 6. Date: August 15, 2019.

## Background

### Epidemiologic transition in Vietnam, cardiovascular disease, and hypertension

Vietnam is undergoing an epidemiological transition with the morbidity and mortality from non-communicable diseases having risen rapidly over the last several decades [1, 2]. This transition can be attributed to changes in population size, socio-demographic characteristics, and increases in life expectancy [1–4]. Cardiovascular disease (CVD) is now the leading cause of death in Vietnam, accounting for 30% of all deaths annually in 2010 [5]. Major risk factors for CVD including hypertension, diabetes, unhealthy dietary practices, and overweight/obesity are either on the rise or at alarming levels in Vietnam [2, 6, 7]. National data showed that the prevalence of hypertension (HTN) was more than 40% for those 50–69 years old and the general population consumed high levels of sodium in their diet in 2016 [7]. Although antihypertensive medications are off-patent, widely available across the country, and are covered by public health insurance (more than 80% of Vietnamese have health insurance), the awareness and management of hypertension are far from optimal [8]; this is due to many factors including lack of regular screening and patient self-management strategies (e.g., medication adherence, lifestyle modifications). Inasmuch, the burden of CVD will continue to increase in the Vietnamese population unless effective prevention and control measures are put in place.

The health care system in Vietnam is organized into four levels, which include the central, provincial, district, and commune (community) level. The lowest level includes the community health centers (CHCs), which are responsible for providing primary health care and outpatient services, including implementation of national health programs. There is typically one CHC per community, and at each CHC, there are approximately 10–15 community health workers (CHWs). These individuals are “natural helpers” without medical degrees who serve as health advocates for their community [9], educators, and problem-solvers, and they provide valuable linkages to available community resources [10]. Recent studies have documented the positive role of CHWs in improving HTN control through better home monitoring, appointment keeping, medication adherence, and health care use [11–13]. CHWs are present in our partnering rural clinics and were an integral part of our previous work [14–16].

Between 2014 and 2016, a feasibility cluster trial of a storytelling intervention was conducted in Hung Yen province, Vietnam, by members of the study team [14–16]. The study included 160 patients with HTN with a mean age of 66 years and 54% were men. Between baseline enrollment and the 3-month follow-up, systolic blood pressure (BP) declined by 8.2 mmHg (95% CI 4.1–12.2) in the storytelling group and by 5.5 mmHg (95% CI 1.4–9.5) in the comparison group; HTN medication adherence increased in the storytelling group and declined in the comparison group. Building on the findings of this feasibility trial, we proposed a full-scale cluster randomized controlled clinical trial to evaluate the implementation and effectiveness of two multi-faceted community and clinic-based strategies on the control of elevated blood pressure among adult men and women in Vietnam.

## Methods

### Intervention approaches and implementation framework

#### Patient-centered interventions

Interventions focused on how patients may reduce their risk of CVD through lifestyle changes including weight control, increased physical activity, tobacco avoidance, moderate intake of salt and alcohol, and adherence to prescribed medications [17–19].

#### Home blood pressure self-monitoring

Home self-blood pressure monitoring enhances patient self-management and empowerment. Compared with office-based readings, home blood pressure monitors may provide better prognostic information about CVD outcomes [20]. Several studies have documented the value of home blood pressure monitoring in improving HTN control [21–23] and increased patient adherence to blood pressure lowering medications [24, 25].

#### Community health workers

Community health workers (CHWs) are “natural helpers” who serve as health advocates for their local communities [9]. A CHW may serve as an educator and problem-solver and provide a valuable linkage to community resources and health promotion activities [10, 26]. Recent studies have documented the positive role of CHWs in improving HTN control [11, 12, 27], and CHWs are present in our partnering rural clinics and were an integral part of our previous work [14–16].

#### Intervention model

**Fig. 1:**
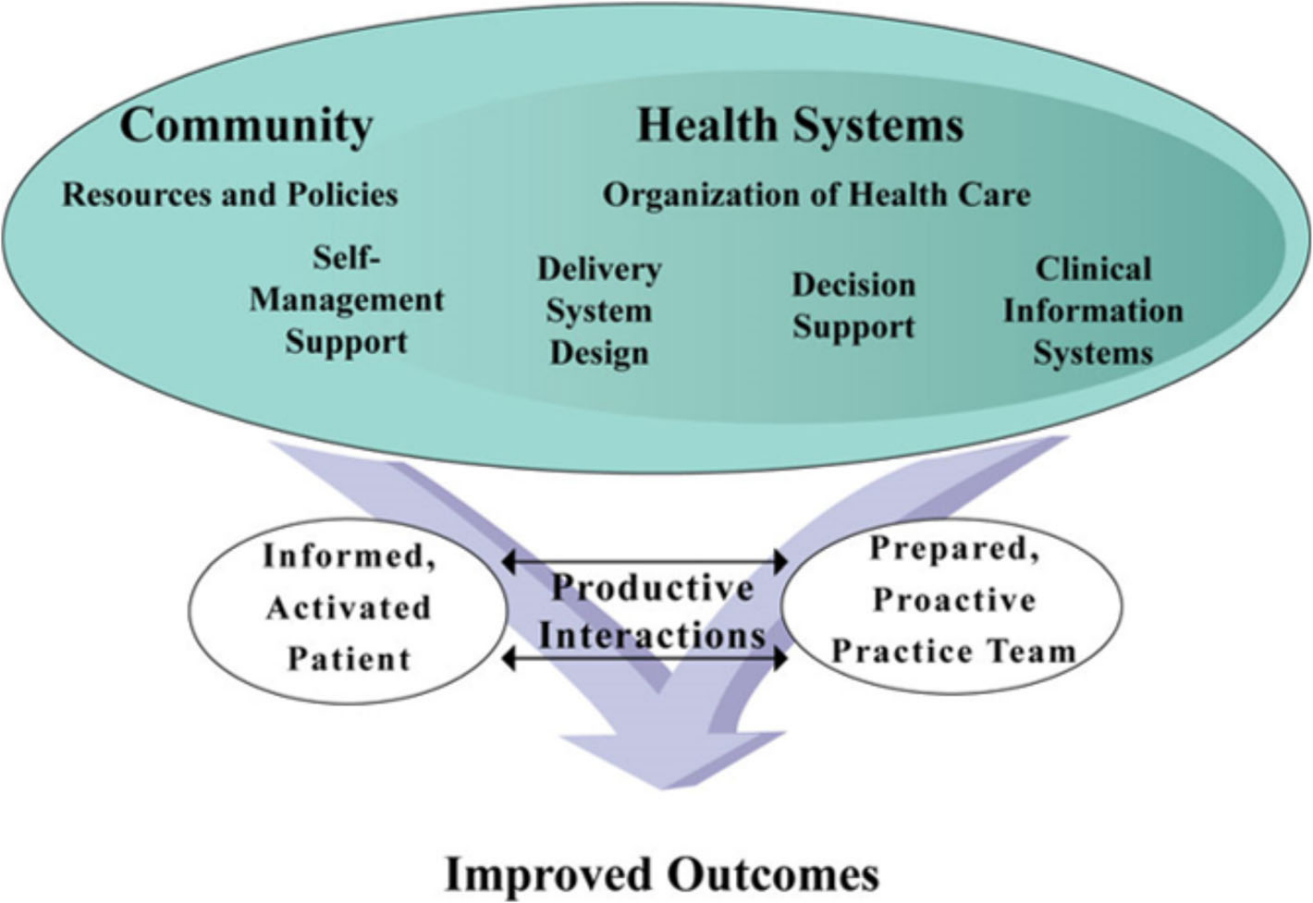
The adapted Chronic Care Model

Wagner’s original Chronic Care Model [28], subsequently expanded [29], provides a conceptual foundation for addressing multi-level barriers to HTN control (Fig. 1). Although we do not have the capacity in rural Vietnam to implement all components of this model, it still provides a systematic framework for organizing our multi-level intervention. This model has been successfully used for HTN management [12, 30, 31], and we have adapted it for the rural Vietnamese setting to guide our intervention strategy. Our intervention components map directly to the Chronic Care Model in the domains of patient self-management support (storytelling, home blood pressure monitoring), clinician decision support (medication management as part of the Vietnam National HTN Program), delivery system design (standardized blood pressure measurement), clinical information systems (tracking software available through the Vietnam National HTN Program), health care organization (leadership buy-in), and community resources (CHWs).

#### Implementation framework

While we considered several implementation frameworks [32–34], we chose the Promoting Action on Research Implementation in Health Services (PARiHS) model because we will be implementing a multi-level model in a de-centralized system with semi-autonomous clinical delivery systems. PARiHS relies on three interactive elements: *evidence*, *context*, and *facilitation* [35–37], which play key roles in our intervention.

Our specific aims are as follows:

1. Conduct a pre-implementation local needs assessment and formative planning in 16 partnering communes in Hung Yen province, Vietnam, leading to a context-specific protocol for implementing the Vietnam National Hypertension Program in both intervention and comparison communities and proposed enhancements (expanded CHW services, home blood pressure self-monitoring, and storytelling in the 8 intervention communities only).
2. Implement a cluster randomized controlled trial (RCT) (Type I Hybrid Implementation Design) of 16 communities and 680 patients with HTN randomized to either an intervention group (Vietnam National Hypertension Program plus 3 trial enhancements) or a comparison group (Vietnam National Hypertension Program alone).
3. Compare the effectiveness and implementation success of the two approaches using data from multiple sources at multiple points in time, including blood pressure measurements, patient surveys, and interviews with clinic personnel and clinicians. Our main study hypothesis is as follows: at 12 months post-randomization, participants in the intervention group will have a greater mean reduction in their levels of blood pressure than those in the comparison group.

### Study setting

The study will be conducted in the Red River Delta Region in northern Vietnam. In this region, communes (communities) in Hung Yen province were selected based on their general representativeness. Hung Yen province has a population of approximately 1.3 million, organized into 10 districts and 161 communes. In Vietnam, the health system is organized into four levels, namely central, provincial, district hospital, and the lowest level, which includes the community health centers (CHCs) that are responsible for providing primary health care and outpatient services. Patients with HTN are typically treated and managed at the district health centers unless they need to be referred for a higher level of care.

### Needs assessment study

During the first 6 months of funding, we will conduct a qualitative study—needs assessment survey at participating study sites. We recognize the multi-level ecological context of our intervention with layers of influence on health status, health behaviors, and behavioral changes beyond the individual. These include (1) community (individuals and families), (2) participating organizations, (3) socio-cultural environment, (4) physical built environment, and (5) the broader policy environment.

The needs assessment survey will be based on the triangulation of multiple data sources, including databases documenting the prevalence and control of HTN in the study communes, and semi-structured interviews with clinicians, clinic staff, CHWs, and community members. The structured interviews will ascertain perceptions of clinicians and clinic leadership about the evidence for treating HTN (evidence), strengths and limitations of the current environment for implementing new tools for HTN control proposed as part of our intervention (context), and specific approaches needed to overcome barriers to blood pressure control (facilitation).

As part of the needs assessment survey, we will perform 21 full semi-structured, individual interviews in a randomly selected subset of three communities from the intervention sites and three communities from the comparison sites. This will consist of semi-structured interviews with clinicians, nurses, and leadership at the health centers and interviews with patients with uncontrolled HTN. We will work closely with the Department of Health in Hung Yen province to identify stakeholders who understand and are involved in HTN management in the community and physicians who have managed HTN patients at the provincial and district hospitals to participate in the study. Patients with uncontrolled HTN will be referred by their physicians at the local hospitals. Brief structured qualitative assessments will be conducted at all remaining sites via focus group discussions (FGDs). We will conduct 9 FGDs at 3 study communities.

These interviews will be repeated on three occasions in study years 1, 3, and 5. The second and third rounds of interviews will employ a similar design to the first round, and there several additional questions will assess the progress of the study implementation. We anticipate that after intervention implementation, the gaps in HTN management found in the needs assessment study will be narrowed to a greater degree in the intervention group. Information on intervention acceptability, appropriateness, feasibility, and fidelity will be collected as well.

### A cluster randomized controlled trial (Type I Hybrid Implementation Design)

**Fig. 2:**
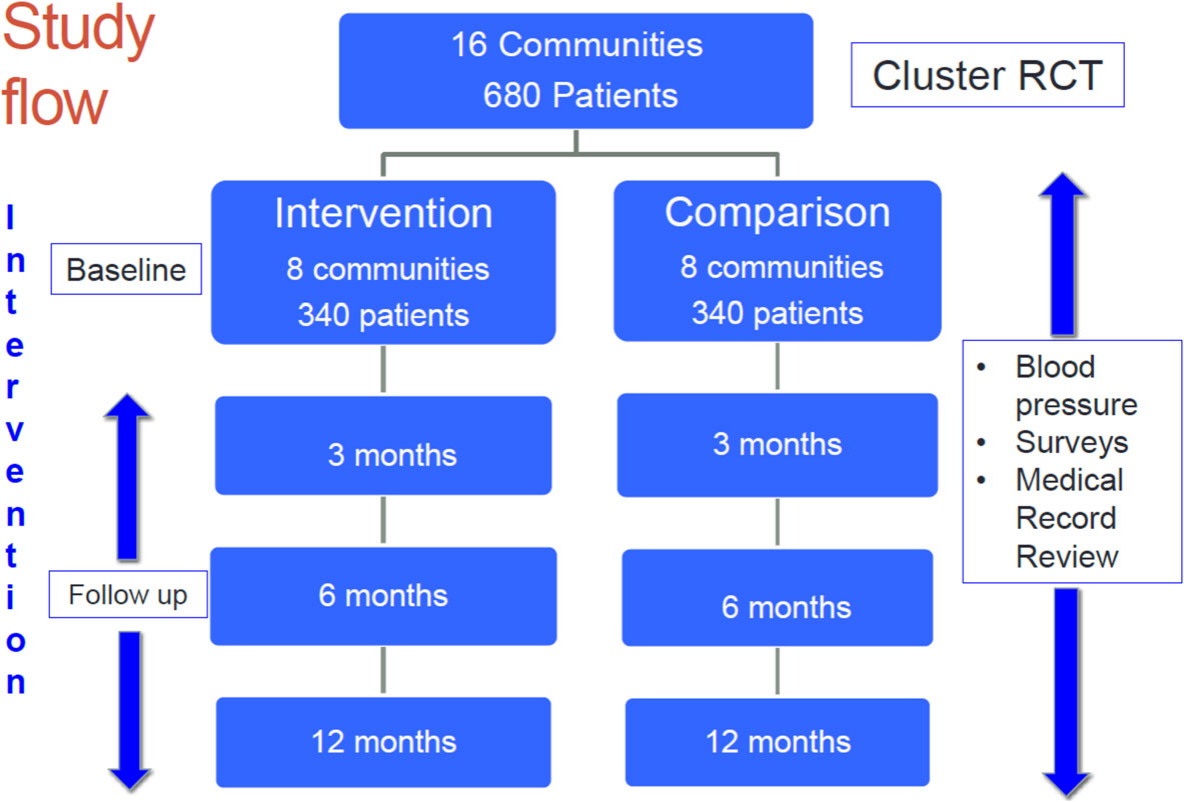
Study flow

**Fig. 3:**
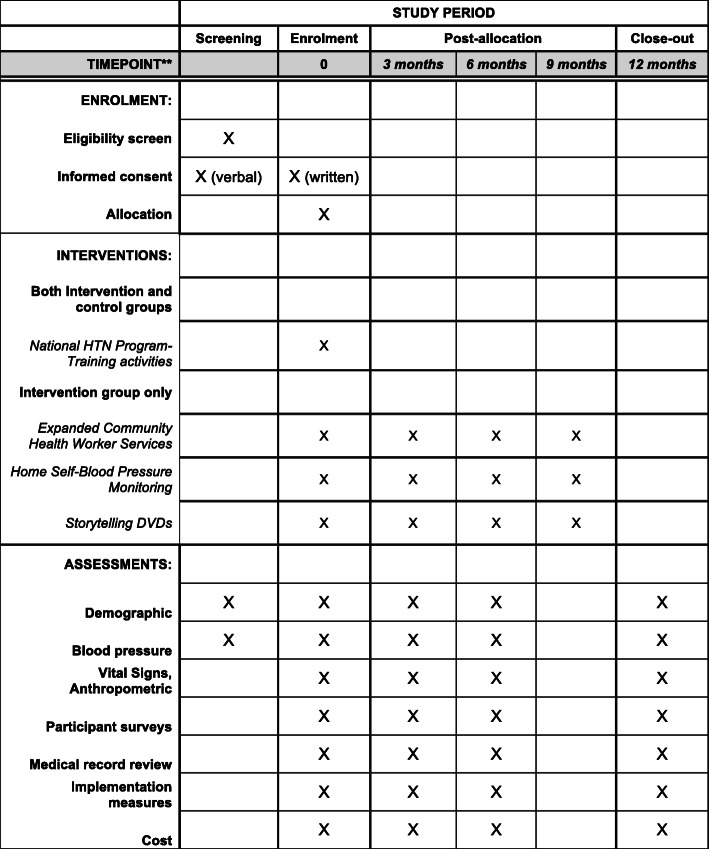
The schedule of enrolment, interventions, and assessments

A full-scale cluster RCT will be conducted in Hung Yen province, Vietnam. Sixteen communities (communes) and 680 patients with HTN will be randomized to either an intervention (Vietnam National Hypertension Program plus 3 trial enhancements) or a comparison group (Vietnam National Hypertension Program alone) (Fig. 2). The schedule of enrolment, interventions, and assessments is presented in Fig. 3.

#### Study sites

A total of 16 eligible communes in 4 districts in Hung Yen province will be randomly assigned to either the intervention (*n* = 8) or the comparison group (*n* = 8). Each of the selected communes satisfies the following criteria: (1) have a CHC with a medical doctor, (2) are not currently participating in other studies for HTN control, and (3) have a minimum geographic separation of 12 km (7 miles) from all other study communities to minimize possible contamination. Eligible and consenting trial participants with uncontrolled HTN (*n* = 680; approximately 43 patients per commune) will be assigned to intervention versus comparison status based on the community in which they reside.

#### Participant eligibility

To be enrolled in our pilot study, consenting adult men and women must fulfill each of the following criteria: (1) be a resident of the selected commune and have no plans for moving during the next 12 months after trial enrollment; (2) be aged 25 years or older, a cutoff which is commonly used in population-based surveillance studies of HTN in Vietnam [6, 8]; (3) have a diagnosis of uncontrolled HTN according to the 8th Joint National Commission of High Blood Pressure (JNC 8) [38]; (4) not be cognitively impaired (as assessed by study physicians); (5) not be a “story teller” used to develop the intervention; (6) not be a family member of another participant in the study; and (7) not pregnant.

In addition, those diagnosed with elevated BP for the first time will be invited for re-measurement of their BP over the next 2 weeks (minimum of 1 week apart) after their initial CHC visit. If their average BP remains elevated, these persons will be invited to participate in the trial. Trained study nurses at study sites will obtain informed consent from eligible patients. Both newly diagnosed and prevalent patients with HTN (treated or otherwise) will be enrolled. If they are not treated at the time of trial enrollment, they will be referred to their district health centers for a follow-up exam and initiation of treatment, which will be covered by public health insurance. Members of the study team will work closely with doctors at the district health centers to follow up these patients.

#### Study recruitment and randomization

Sixteen communities will be randomly assigned to either the intervention or the comparison condition stratified by districts using the STATA program. In each district, 2 communes will be randomly assigned to the intervention group and 2 communes will be randomly assigned to the comparison group. Patients will be recruited from the community setting. Screening events to identify patients satisfying our study criteria will be conducted at local CHCs. A second screening visit will be scheduled for eligible patients with elevated blood pressure (BP) values (systolic BP ≥ 140 mmHg, or diastolic BP ≥ 90 mmHg) 2 weeks later, at which time their BP will be re-measured and written informed consent will be obtained. Individuals who are found to have elevated BP at the time of clinic screening and are not willing to participate in the study will be referred for usual care at local district health centers.

The recruitment will rely on CHWs embedded in both the clinic and the community, which will provide credibility and trust. The CHWs and study nurses will offer BP screenings at their CHCs. With this approach, our recruitment goals will achieve in the allotted time.

After enrollment, a reminder letter will be sent to all participants before each of the follow-up visits. One week before the scheduled follow-up visit, local staff will contact study participants by phone. Because the communes are small, it is easy to visit participant’s homes. If patients miss their follow-up visit, local staff will contact them by phone to remind them to come into the CHC or visit their homes to measure their BP, if necessary. Patients can withdraw from the study at any time during the study period. If patients move out from their communes permanently, they will be considered as dropping out from the study.

#### Intervention condition and delivery

Detailed interventions and delivery methods are described in Table 1.

**Table 1:** Intervention components by study group

	Intervention enhancements	Intervention	Comparison
**1**	**Vietnam National HTN Program** Training for physicians and nurses Patient education materials Multi-media community announcements	Yes	Yes
**2**	**Expanded community health worker services** Support and strengthen their role in motivating patients through lifestyle changes and antihypertensive medication adherence	Yes	No
**3**	**Home self-blood pressure monitoring** Free home BP monitors Record BP in a pre-tested log and share with their physicians and CHWs	Yes	No, will be given BP monitor and BP log after the study has ended
**4**	**Storytelling intervention** 4 DVDs with stories from Stars: baseline and 3, 6, and 9 months Learn More Module	Yes	No (only 2 DVDs with Learn More Module), will be given 4 storytelling DVDs after the study has ended

##### Vietnam National HTN Program (intervention and comparison groups)

The Vietnam National HTN Program is part of a comprehensive Vietnam National Strategy on Prevention and Control of Non-Communicable Diseases [39]. This multi-arm national strategy was approved by the Prime Minister in 2015, merging several national programs into a cohesive, integrated approach. The Vietnam National HTN Program was authorized in 2008 including various training sessions about HTN prevention and management for physicians and nurses, and a comprehensive set of patient education materials written in a culturally and literacy-appropriate manner. Multi-media community service announcements have been prepared for local television and radio stations and newspapers, and will be implemented in both study groups. There will be a series of training sessions for health care providers which will be carried out at local district or provincial health departments in collaboration with the Vietnam Ministry of Health.

##### Expanded community health worker services (intervention group only)

CHWs are currently embedded in the clinical system for each of our partnering CHCs and across the nation. A critical enhancement for the intervention group will be to support and strengthen their role in activating patients to more actively manage their HTN through lifestyle changes and adherence to prescribed antihypertensive medication. CHWs will be trained in motivational interviewing and structured problem-solving. This behavioral change counseling approach facilitates improvements in diet, exercise, adherence to medication regimens, tobacco use, and overall engagement in one’s care [40]. CHWs without advanced health care training can be safely trained in this dynamic approach to facilitate health behavior change with proven effectiveness [41, 42].

CHWs will also be taught simple techniques to help patients set goals for a number of lifestyle changes, including salt and alcohol reduction, smoking cessation, increased physical activity, and enhanced medication adherence; they will also develop problem-solving strategies to achieve these goals. CHWs will be taught how to work with patients to (1) engender engagement and commitment by self-identifying goals that are meaningful and consistent with their personal lives and family context; (2) promote feasibility by identifying a limited set of goals and small, attainable steps; (3) provide educational resources; (4) establish a structure for accountability and support through regular review; and (5) link goal attainment to changes in self BP monitoring for reinforcement. These techniques were successfully used in our pilot work [15].

As in our pilot work, CHWs will be taught how to use the storytelling intervention (below) to start conversations with their patients with elevated BP values. After each DVD of the storytelling intervention, CHWs will meet with the patient to review the material, elicit possible barriers to lifestyle changes and medication treatment, and identify strategies to overcome recognized barriers. CHWs will make bi-weekly patient home visits (1 h) to resolve difficulties related to viewing the DVDs, and they will keep detailed logs of their patient interactions to help provide a qualitative sense of intervention effectiveness and suggest approaches for improvement. There will be a series of training sessions for CHWs, which will be delivered locally in the collaboration with the Vietnam Ministry of Health.

##### Home self-blood pressure monitoring (intervention group only)

Home BP self-monitoring is the second enhancement for the intervention group. The intervention group will receive free home BP measurement devices at the time of trial enrollment at their CHCs whereas patients in the comparison group will receive home BP monitors and a BP log after the study has ended. After obtaining informed consent, a trained CHW will instruct patients on how to use the BP measurement devices at home and how to record their BP readings in the BP log previously developed and implemented by the study team. Readings will initially be taken in the morning after arising and again at night before going to sleep. Patients will be advised about reading variability, cautioned about overreacting to a single elevated BP value, and given specific protocols for when to contact a health care provider should the need arise. Patients are routinely given a portable copy of their medical record with instructions to bring it to future clinic appointments.

##### Storytelling intervention (intervention group only)

Our team has previously designed, implemented, and pilot tested such an intervention for improving HTN control in Vietnam [14–16]. The patient narratives included in the intervention materials include first-hand accounts from patients in their journey to gain control of their HTN, and the stories are complemented by additional formal information about HTN control. This Learn-More section will be built on the stakeholders’ opinions gathered via interviews and national experts in HTN control. For our newly proposed work, we will supplement this previously developed material with new patient stories to represent our expanded community base. We will develop four storytelling DVDs, the first to be delivered to intervention patients at the time of trial enrollment, with viewing in the clinic, followed by installments at 3, 6, and 9 months to be viewed at home. All intervention participants will be provided with a DVD player and instructed in how to navigate the menu structure of the DVDs at enrolment. After each installment, we will administer a post-media interview to ascertain the frequency and duration of viewing, change in behavioral intentions, and overall satisfaction with the intervention; these data will be used for the mediation analysis to describe implementation and mechanisms of intervention effectiveness. At 3 and 6 months after trial enrollment, a second and third installment of the DVDs will be delivered at the patient’s local CHC for home viewing by patients assigned to the storytelling intervention group. At 9 months after trial enrollment, the fourth DVD will be delivered at participant’s home by the CHWs.

We will develop 2 short DVDs with Learn-More section only for the comparison group. Patients in the comparison group will receive a DVD player and the first DVD at trial enrollment and the second DVD at month 6 at their local CHC.

For both groups, after viewing the DVDs, a follow-up visit will be scheduled for a “post-media” interview and re-measurement of patient’s BP by a trained CHW.

### Sample size

Sample size calculations are based on our primary trial hypothesis with between-group differences in over-time changes in systolic BP as the principal trial outcome. Our previous pilot work in rural Vietnam suggests that it is feasible to achieve an over-time improvement of 8 mmHg in systolic BP with a standard deviation of 18 mmHg. Analysis of pilot data revealed an intra-class correlation of 0.011 for the clustering of participants in communes for change in systolic BP. We first performed unadjusted sample size calculations that did not account for clustering of individuals within study site and did not inflate for possible losses to follow-up. For these calculations, we set alpha error at 0.05 and examined a range of power from 0.8 to 0.9 based on the two-sided *t* test with a common standard deviation of 18, assuming that the mean improvement in systolic BP is 8 mmHg for patients in the intervention condition and 3 mmHg for those in the comparison condition.

Next, we adjusted these first-pass sample size calculations to account for the clustering of participants. According to Donner: *N*_adjusted_ *= N*_undjusted_(1 + (*m* − 1)*r*), in which *N*_adjusted_ is the total sample size adjusted for clustering, *N*_undjusted_ is the unadjusted total sample size, *m* is the unadjusted average cluster size (average number of patients/community), and *r* is the intra-class correlation (ICC) [43]. Finally, we inflated the resulting sample size by approximately 10% to account for potential losses to further follow-up. It is important to note that the planned analyses for this study will draw upon the power of longitudinal measurement, which will be more powerful than the above-presented estimates [14]. Data to inform these calculations were based on recently published work and are summarized in Table 2.

**Table 2:** Sample size calculations

Power	Sample size		
	Unadjusted	Cluster-adjusted^1^	Cluster-adjusted and retention inflated^2^
0.80	410	522	573
0.85	468	614	**674**
0.90	548	549	823

Sample size calculations assume an improvement in systolic blood pressure of 8 mmHg in the intervention group and 3 mmHg in the comparison group for a differential, over-time improvement of 5 mmHg. Alpha error is set at 0.05

^1^According to approach described by Donner and setting intra-class correlation coefficient at 0.011 based on pilot data

^2^Final calculations are inflated by approximately 10% to account for potential loss to follow-up

In addition to the main analyses described above, we also anticipate adequate power for the planned mediation analysis. For the mediation analysis, simulation studies revealed that a sample size of 500 is adequate to detect pathways with small standardized effect sizes (as low as 0.14) at 80% power with methods described above [44].

#### Data collection

Data sources include the following: (1) standardized BP and anthropometric measurements at baseline and at 3, 6, and 12 months after trial enrollment; (2) quantitative participant surveys at baseline and at 3, 6, and 12 months after enrollment; (3) post-media interviews after each installment of the storytelling intervention for the intervention group; (5) medical record review; (6) implementation data gathered by the research coordinator and CHWs that will inform progress toward specific study milestones; and (7) semi-structured interviews for qualitative data.

##### Blood pressure and anthropometric measurement

As previously described, certified study nurses will be trained to measure BP according to a standardized protocol approved by the World Health Organization [45]. We used this protocol in our previous randomized trial of storytelling in Vietnam. This protocol was written for use with the *OmROn* HEM-8712 automated BP monitor, with special attention to assessment and maintenance of the instrument’s accuracy and training/certification of research assistants. Using a proper cuff size, measurements are taken after sitting quietly for 5 min, with the arm supported on a flat surface, with the upper arm at heart level. Three measurements are separated by at least 1 min, and values from the last two measurements will be averaged. Height and weight will be measured in the absence of shoes and heavy clothing while waist and hip sizes will be measured by placing the tape horizontally around the smallest part of the waist and the widest portion of the hips, respectively.

##### Participant surveys

All survey items will be taken from validated instruments, with scales or sub-scales left intact to preserve psychometric properties [46]. The survey will be implemented in a computer-assisted format and pilot tested for acceptability. The target duration time for the final survey will be less than 1 h. We will collect information on patient’s level of education, occupation, and economic circumstances using the WHO STEPs protocol [47, 48]. STEPs will also be used to collect data on CVD risk factors including tobacco use, alcohol consumption, salt intake, and physical activity. Adherence to anti-HTN medications will be measured using standardized forms previously developed by Duke University [49]. The Medication Adherence Self-efficacy Scale (MASES) instrument [50, 51] will measure self-efficacy in HTN management. Quality of life will be measured by the short form 12 questionnaire health survey (SF-12) [52]. All the survey measures previously existed in Vietnamese or have been translated and tested by our team as part of our previous work [15] with the exception of the adherence to anti-HTN medications, which will be translated following the set of best practices developed by the US Census Bureau [53]. Translation will be accomplished by a translation team led by study PIs, with multiple versions prepared in parallel followed by team meetings to reconcile possible differences. This approach has been shown to be superior to the simple “back-translation approach” in which translation by a single individual is translated back into the source language for review of accuracy [54]. Translation will include cognitive interviews with five participants drawn from the local community. Cognitive testing will identify constructs specific to the Vietnamese language and culture so that appropriate adjustments may be made to ensure cross-cultural equivalence [55]. Several cycles of revision will be accomplished at full translation committee meetings conducted in person and by Internet video link.

Post-DVD viewing interviews for intervention participants will be based on protocols previously developed by our team. The post-DVD viewing interviews using a structured questionnaire will collect self-reported engagement with the DVDs, including total viewing minutes, specific segments that were viewed, and whether the DVD was shared with family or friends. “Transportation” is a validated concept measuring absorption into the video story that has been linked to intervention effectiveness and is measured by a validated scale [56, 57]. Participants will be asked to elaborate on what motivated/hindered their intervention engagement.

#### Data management

Data will be stored on a Health Insurance Portability and Accountability Act (HIPAA)-compliant secure server with daily backup at the Health Strategy and Policy Institute and managed by the study data manager who is familiar with the REDCap database. This person will work closely with the PIs and experts at University of Massachusetts Medical School to make sure that the data are managed properly.

#### Study outcomes

##### Primary outcomes

Change in patient’s systolic BP levels over the 1-year follow-up period is the primary trial outcome. Registered nurses will be trained and certified to measure patient’s BP according to a protocol approved by the World Health Organization [45]. Three measurements of BP will be carried out, and values from the last two measurements will be averaged with the first reading ignored.

##### Secondary outcomes

Changes in diastolic BP, risk factors for CVD, medication adherence, self-efficacy, quality of life, cost, and implementation outcomes are secondary outcomes of this study. The WHO STEPs survey, which has been used to investigate the epidemiology of HTN in the Vietnamese language [48], will be used to collect data on several risk factors for CVD. Costs include the following: (1) program costs, which consist of costs to develop the intervention and implementation costs incurred at the district and community levels, and (2) patient costs such as drugs, diagnostic procedures, time lost, health center visits, and consultation fees.

Implementation outcomes including acceptability, appropriateness, adaptation, feasibility, fidelity, and sustainability will be collected via semi-structured interviews and focus group discussions among stakeholders and patient interviews, and post-DVD viewing surveys at follow-up visits. The implementation data and barriers to study milestones achievement gathered by the research team will inform strategies to overcome barriers and progress toward specific study milestones.

#### Data analysis plan

##### Qualitative analysis

In particular, we will use a well-accepted type of qualitative analyses often described as thematic analysis. Thematic analysis will be applied to the narratives obtained via Story Development Groups as well as from individual interviews from Video Stars and will proceed as follows: (1) entering transcripts into NVivo (qualitative software), (2) performing initial in vivo coding to separate the full story into initial story units, (3) tagging story units with codes from pre-existing codebook as well as any additional themes that emerge, and (4) grouping related codes based on broader themes connecting the experiences of participants to key concepts from our conceptual frameworks, which will be explored in the intervention. Thus, our approach to thematic analysis will include open coding, followed by concept building, and the development of themes/categories [58, 59]. Three members of the team will code these data—using the Link and Phelan framework [60] as a guide. Rich data will be gathered from multiple sources to drive the qualitative analyses. Interview notes and recordings from the Story Development Groups and interviews of Video Stars will be linked by a code, and recordings will be destroyed after verification of notes.

For needs assessment study, we will also utilize thematic qualitative analysis utilizing the qualitative software platform NVivo to objectively identify and catalog the perceptions expressed in these interviews. The interviews will be analyzed utilizing a thematic analysis approach to identify central themes as they appear in interviews. The identified overarching themes will serve as a baseline to build upon the packaged narratives to be presented visually in the DVDs.

Detailed field notes and codebooks will be maintained. Qualitative data analysis software will support the coding activities for the thematic analysis process above and will allow for inter-rater reliability testing among multiple coders, who will achieve at least a 90% agreement rate.

##### Quantitative analysis

We will begin the statistical analysis by examining univariate statistics, including measures of central tendency and dispersion. We will carefully document the trial recruitment and retention process with a CONSORT diagram [61, 62]. In accordance with best practice, differences in baseline characteristics of the intervention and comparison groups will be established based on standardized differences, rather than on tests of statistical significance [63, 64].

All primary hypothesis testing will be performed on an intent-to-treat basis and will be two-sided with alpha error will be set at 0.05. For the main study hypothesis, the continuous outcomes will be systolic (H1) and diastolic (H2) BP. We will use a generalized linear mixed model adjusting for important potential covariate imbalances between the two primary study comparison groups. Since we will collect longitudinal data with repeated observations nested within participant, and participants nested within community, many statistical analyses will be based on a generalized linear mixed model with Restricted Maximum Likelihood (REML) estimation that accounts for the complex data structure through random effects response [65–68].

We will use mediation analysis to disentangle the multiple mechanisms which may be associated with the effectiveness of our multi-level intervention. Patient-level mediators of intervention effectiveness include the following: (1) intervention engagement (measured by number of CHW sessions completed and time spent in the sessions, types of goals set and corresponding action plans, BP self-monitoring, and engagement with the storytelling intervention), (2) medication adherence, (3) adherence to heart-healthy lifestyle recommendations, and (4) patient activation. Clinic-level mediators include implementation of standardized BP measurement protocols, engagement of physicians and nurses in the trial educational programs, and fidelity of CHW intervention delivery.

For the secondary analysis, we will examine differential over-time changes in diastolic BP, HTN control, and CVD risk, using statistical approaches described above for testing the main study hypothesis. For these analyses, a dichotomous measure of BP control will be constructed according to JNC-8 [38]. Overall risk of a CVD event at 10 years will be calculated based on the Asian Pacific Cohort equation [69]. We will examine changes in patient’s individual risk factors for CVD (e.g., smoking, physical inactivity) as well. We will carry out exploratory analyses for possible heterogeneity of intervention effect among sub-groups of participants defined by age, sex, and existing CVD.

Missing data may introduce potential bias, and the most important defense to minimize missing data on key factors is advanced planning [70, 71]. In our previous work, we have developed successful plans to maximize participant retention and obtain complete data through sound principles of data collection and quality control [15]. Sensitivity analyses will estimate the bounds of potential bias introduced by omissions. Under the missing-at-random assumption, multiple imputation [72] will generate plausible values of missing covariates while accounting for the additional uncertainty introduced by the omissions. All the analyses will be performed using STATA 16.0 (Stata Corp, TX).

The economic analysis will be led by Dr. Ha, who has expertise in evaluating the cost-effectiveness of interventions to prevent CVD in Vietnam [73]. From the societal perspective, we will analyze costs and effectiveness for the intervention package with the proposed enhancements in comparison to standard implementation of the Vietnam National HTN Program, using approaches appropriate for lower-to-middle income countries [74, 75]. Costs include the following: (1) program costs, which consist of costs to develop the intervention and implementation costs incurred at the district and community levels, and (2) patient costs such as medications, time lost, health center visits, and consultation fees. We will calculate the incremental costs of the intervention by subtracting the average costs for the intervention group from the average costs for the comparison group. The incremental cost-effectiveness ratio will be calculated as the additional cost of the intervention divided by the change in both systolic and diastolic BP related to the intervention. In addition, we will calculate cost-effectiveness ratios for BP control, by dividing the additional costs of the intervention by the proportion who achieved HTN control as a result of the intervention. Because this is a country-specific economic analysis, all costs will be evaluated using the Vietnamese Dong (VND) with subsequent conversion to US dollars. We will perform one-way sensitivity analyses related to variable intervention effectiveness, variable engagement, and trial adherence.

#### Timeline

The proposed study will take place over a 5-year period. During the first 6 months of year 1, we will conduct a needs assessment study. The next 18 months will be devoted for intervention enhancement development. Training for intervention delivery will be conducted in early year 3, right before the trial start (year 3). All data collection activities for the trial will be completed by mid-year 5. Year 5 will be devoted to data analysis and manuscript writing.

#### Trial oversight

The trial steering committee is composed of all PIs and study key personnel from all sites. This committee is responsible for designing and implementing the study and recommends appropriate actions to ensure that protocol deviations will be minimized. An independent Data Safety Monitoring Board (DSMB) has been established to be responsible for safeguarding the interests of study participants, assessing the safety and efficacy of study procedures, and monitoring the overall conduct of the study. Communication with DSMB members will be primarily through the DSMB administrator (Executive Secretary—ES) and the Data Coordinating Center (DCC). The first DSMB meeting will be held before the trial start to review study protocol and give approval and will meet every 6 months to reviewing data for safety and feasibility. Additional meetings will be arranged as needed. An interim analysis will not be performed. The DSMB may make recommendations whether the study/the trial will be continued or stopped. The DSMB will also receive all Serious Adverse Event (SAE) reports and may request additional information, as needed. In addition, on-site monitoring visits from a qualified research monitor will be scheduled quarterly until data quality is deemed acceptable and then will be scheduled 6 monthly for the remainder of the study. The study protocol conforms to the SPIRIT checklist (Additional file 1).

#### Dissemination plan

The project will work closely with policymakers from the Vietnam Ministry of Health to transform evidence into a policymaking process as well as with the Vietnam Heart Association to scale up the interventions and implementation program and make them more widely available. The process will start with a dissemination workshop and then forums for policy dialogue with participation of key leaders and influential people from the Ministry of Health, policymakers, health managers, practitioners, and stakeholders who are involved with the prevention and control of non-communicable diseases in Vietnam. The results of the trial will be published in international peer-reviewed journals by the study team.

## Discussion

We are proposing a full-scale cluster RCT to test the effectiveness of two strategies to improve HTN control for adults residing in rural communities in Vietnam. We will use a well-planned approach to culturally adapt intervention enhancements that have been effective in our feasibility trial and from other settings. If our approach is proven to be successful, it will offer policymakers an innovative intervention strategy to address a well-recognized and emerging threat to public health in Vietnam. Our approach is built on many previous “lessons learned” and, more importantly, is low cost and of low burden to patients, clinicians, and health care systems.

However, there are some potential study caveats. Due to the nature of the study design, the trial is not blinded. With 12 months of follow-up, we are unable to assess the long-term effects of the intervention on CVD morbidity and mortality. Also, given limitations of sample size, we are not testing each intervention component separately. Still, mediation analysis and analysis of implementation data will help disentangle the contributions of specific intervention components. Another potential threat is contamination of the comparison group, if the storytelling DVDs or home BP monitors are shared. Thus, we specified that the distance between an intervention and comparison community will be at least 10 km. Evidence of contamination will be sought through patient interviews at the final study visit.

Patient recruitment to the trial may lag, or the intervention may not be faithfully implemented. We recognize the need for proactive monitoring and response, and will implement previously developed protocols that were successful in rural Vietnam. Implementation data with specific milestones will provide early warnings to guide corrective action. We will work with the NIH Scientific Officer and the structure for coordination across studies to be funded under the Hypertension Outcomes for T4 Research within Lower Middle-Income Countries (Hy-TREC) initiative for scientific guidance as appropriate and to establish meaningful study milestones.

The “Conquering Hypertension in Vietnam: Solutions at Grassroots Level” will provide new evidence about the effectiveness and implementation of the Vietnam National HTN Program, which is being rolled out across the nation but has not been part of a randomized trial in its current format, and will evaluate our proposed enhancements to this ambitious national effort.

## Trial status

The recruitment began on October 23, 2019, and will complete on March 1, 2021.

To date, randomization has been finished and 423 patients have been recruited from 10 communes (updated on October 18, 2020). Ninety-seven percent of patients completed the 3-month follow-up contact (211/218 eligible patients), and nearly 90% have completed the 6-month follow-up contact (84/94 eligible patients). This protocol is version 6, dated August 15, 2019. Any protocol modifications will be communicated to relevant parties (e.g., IRB, trial participants) and published on relevant channels (e.g., ClinicalTrials.gov).

## Supplementary Information

**Additional file 1.** SPIRIT 2013 Checklist: Recommended items to address in a clinical trial protocol and related documents.

## Data Availability

The datasets, informed consent form, and other study materials of the current study will be available from the corresponding author on reasonable request. All data will be de-identified before sharing.

## Abbreviations

BP: Blood pressure
CHC: Community health center
CONSORT: Consolidated Standards of Reporting Trials
CVD: Cardiovascular disease
DVD: Digital video disc
HTN: Hypertension
ICC: Intra-class correlation coefficient
JNC: Joint National Committee
RCT: Randomized controlled trial
WHO: World Health Organization
